# COVID-19 association with purpura fulminans: report of a life threatening complication in a fully vaccinated patient

**DOI:** 10.1093/jscr/rjac095

**Published:** 2022-03-26

**Authors:** Vladislav Pavlovich Zhitny, Mitchell Lyons, Andrea Perloff, John Menezes, Ashley Pistorio, Richard Baynosa

**Affiliations:** Kirk Kerkorian School of Medicine, University of Nevada, Las Vegas, NV, USA; Department of Plastic Surgery, Kirk Kerkorian School of Medicine, University of Nevada, Las Vegas, NV, USA; Department of Plastic Surgery, Kirk Kerkorian School of Medicine, University of Nevada, Las Vegas, NV, USA; Department of Plastic Surgery, Kirk Kerkorian School of Medicine, University of Nevada, Las Vegas, NV, USA; Department of Plastic Surgery, Kirk Kerkorian School of Medicine, University of Nevada, Las Vegas, NV, USA; Department of Plastic Surgery, Kirk Kerkorian School of Medicine, University of Nevada, Las Vegas, NV, USA

**Keywords:** purpura fulminans, hand surgery, plastic surgery, COVID-19

## Abstract

SARS-CoV-2 manifestations have been an ongoing evolving topic that has spread beyond its initial respiratory associations. Recently, there have been reports of COVID-19 infections found to be associated with vascular pathologies. Here, we describe a case of a fully vaccinated COVID-19 adult male with past medical history of purpura fulminans that presented with diffuse necrotic cutaneous tissue sequelae resulting in intensive care unit management and dry gangrene of upper extremity. On admission, it was found that the patient had decreased activity rather than quantity of coagulation pathway protein S. Early recognition and work up are essential in patients with known history of vascular disease and confirmed cases of SARS-CoV-2 positive polymerase chain reaction.

## INTRODUCTION

Since its first report in Wuhan, China, there have been 232 million confirmed cases of COVID-19 worldwide with over 4.7 million deaths [1, 2]. Though the most common SARS-CoV-2 presentation has been its respiratory symptoms, there has been an increasing number of reported skin manifestations noted in the literature with history of vascular pathology [3]. In particular, a rare manifestation has been reported in 2020 for 375 cases in Spain with necrotic cutaneous presentations [4].

Purpura fulminans (PF) is a microvascular coagulation disorder that presents with purpuric lesions and skin necrosis, considered to be a true medical emergency. Patients present with hemorrhage from multiple sites accompanied by disseminated intravascular coagulation (DIC), fever and hypotension [5]. The most common is acute infectious subtype that manifests in septic patients. Varicella is a known viral trigger, with *Streptococcal* and *Meningococcal pneumoniae* species found to be bacterial triggers. Idiopathic PF follows febrile illness after a post-infectious autoimmune sequela where a deficiency of protein S is thought to be the culprit.

Here, we describe the first case in our academic hospital with SARS-CoV-2 necrotic cutaneous presentation found in a patient with history of PF without any other identified etiology in a fully vaccinated adult male.

## CASE PRESENTATION

A 64-year-old male with past medical history significant for hypertension and PF presented to the emergency department complaining of lower back and right hip pain. The patient stated that the symptoms began 2 days prior. He was in too much pain to get out of bed, which he rated at 8/10 denying falls or trauma. He then noticed a discoloration to his low back and hip and swelling on the left leg which prompted him to come to the emergency department. The patient admitted to experiencing flu-like symptoms 10 days prior. He was fully vaccinated for COVID-19 via BioNTech, Pfizer. Review of systems on admission was unremarkable.

On presentation, the heart rate was 96 beats/minute, blood pressure 104/57 mmHg and respirations 25 breaths/minute with SpO2 97%. His physical exam exhibited non-labored respirations without accessory muscle use or audible wheezes. No icterus or injected conjunctiva were observed; the oropharynx was moist without exudates. Extremities were warm and well perfused. There were 2+ radial pulses bilaterally with a full range of motion; 2+ pitting edema was present at the right lower extremity. The skin was warm and dry. Large areas of palpable purpura with surrounding erythema and induration were observed at bilateral buttocks.

Laboratory evaluation revealed elevated lactic acid of 2.43 mmol/l (nl: 0.5–2.2 mmol/l), white blood cell count of 15.66 × 10^9^/l (nl: 4.5–11 × 10^9^/l), blood urea nitrogen of 45 mg/dl (nl: 2.1–8.5 mmol/l) and creatinine of 2.19 mg/dl (nl: 0.74–1.35 mg/dl). Troponin I 78 (nl: 0–22 ng/l), and slightly elevated prothrombin time of 12.5 (nl: 9.3–12.4 s). The patient tested positive for COVID-19 infection via SARS-CoV-2 Real-Time Polymerase Chain Reaction (PCR). Venous duplex ultrasound of bilateral lower extremities showed no evidence of deep venous thrombosis. There was no superficial thrombophlebitis. Chest X-ray showed no obvious focal lung opacities, effusions or pneumothorax. Blood cultures obtained at the time of admission showed no growth. He was admitted to the inpatient ward for a working diagnosis of necrotizing fasciitis vs. cellulitis vs. vasculitis.

On the third day of admission, the patient began to develop superficial skin sloughing along bilateral buttocks; left lower leg ecchymosis persisted with large, non-tense bullae near ankle; left dorsal hand and right elbow ecchymosis were noted. He continued to have an upward trending white blood cell count of 16.69 × 10^9^/l (nl: 4.5–11 × 10^9^/l); however, his blood urea nitrogen level trended down to 21, and creatinine level to 1.14. His Fibrinogen level was 249 (nl: 175–400 mg/dl). The patient tested negative for p-ANCA <1:20 (neg 1 < 1:20), c-ANCA <1:20 (neg 1 < 1:20), myeloperoxidese <9 (nl: 0.0–9.0), proteinase antibody <3.5 (nl: 0–3.5), negative antinuclear antibody <1:80 (neg <1:80), non-reactive HIV-1 and HIV-2 antibodies. Patient’s laboratory showed decreased levels of Protein S activity 26 (nl: 63–140%) with normal Protein S levels of total and free 67 (nl: 61–136%) and normal protein C function 93 (nl: 73–180%) consistent with prior history of PF.

**Figure 1:**
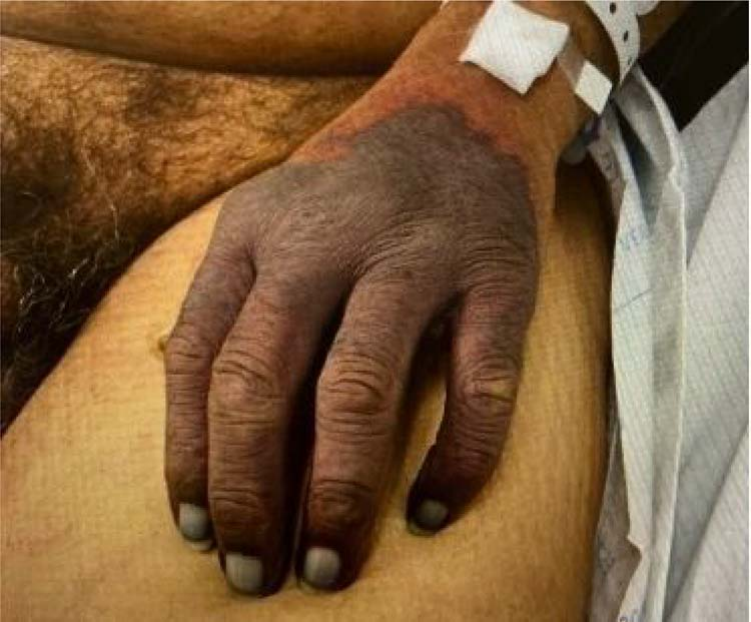
Day 6 of patient admission, necrosis of left hand.

**Figure 2:**
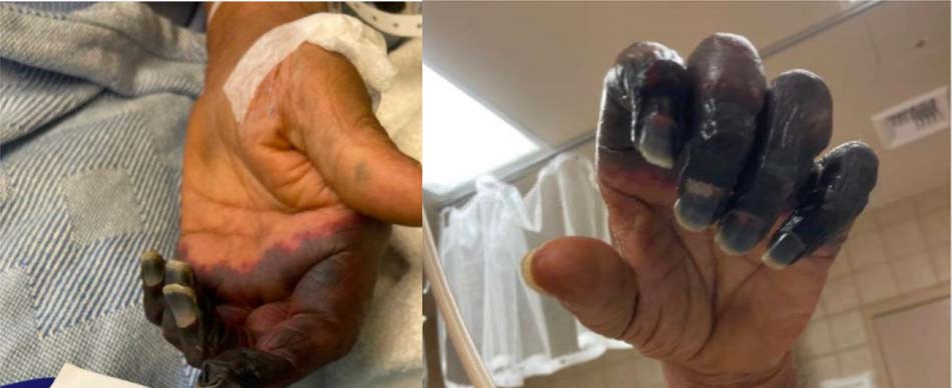
Dry gangrene of the left upper extremity.

On sixth day of admission patient’s d-dimer was severely elevated at 35.20 (nl ≤ 0.5), fibrinogen 50 (nl: 175–400 mg/dl) and a worsening PT of 14.4 nl (9.3–12.4), INR 1.40 (nl: 0.8–1.20). At this time, ICU was consulted for a potentially developing DIC. His upper extremity ecchymosis worsened, now spanning from mid upper arm to fingertips (Fig. 1). Arterial ultrasound of upper extremities showed no arterial thrombus. Patient experienced worsening ecchymoses, for which he underwent extensive debridement by general surgery. Plastic surgery was consulted for continued worsening of left upper extremity which progressed to dry gangrene (Fig. 2). The patient ultimately elected to undergo aggressive debridement of the necrotic tissue including amputation.

## DISCUSSION

Presentations with microvascular thrombosis have been reported as sequela of SARS-CoV-2 [4, 6]. Due to SARS-CoV-2 autoimmune hypercoagulable state, patients with history of vascular disease, such as PF, are found to be at an increased risk for necrotizing skin manifestations, mirroring DIC, termed COVID-19-associated coagulopathy; 4.4% of intensive care unit admitted patients have been found to have arterial thrombotic sequelae, while 27% go on to develop venous thromboembolism [6]. Although the exact mechanisms are yet to be described, some postulate that this may also be due to hypofibrinolysis state due to exhaustion of fibrinolysis [7]. Others go on to describe an imbalance between von Willebrand Factor and ADAMTS-13 axis mirroring pathology similar to thrombocytopenic purpura. There of course are also disease-specific etiologies described as well. Patients with known history of PF are thought to have underlying etiology associated with an imbalance of proteins S and C or antithrombin III [5, 8].

In this case, our patient followed a similar pattern of infectious autoimmune sequelae after 7 days following contact with offending pathogen, believed to be SARS-CoV-2 on work up consistent with reported literature [4, 9]. Our laboratory work up demonstrated decreased levels of Protein S activity with normal Protein S levels. This may lead investigators to look not only into quantity of aforementioned factors but also their activity levels.

## References

[1] Phelan AL, Katz R, Gostin LO. The novel coronavirus originating in Wuhan, China: challenges for global health governance. JAMA 2020;323:709–10. 31999307 10.1001/jama.2020.1097

[2] World Health Organization . (n.d.). Coronavirus disease (covid-19). World Health Organization. https://www.who.int/emergencies/diseases/novel-coronavirus-2019 (30 September 2021, date last accessed).

[3] Levi M, Thachil J, Iba T, Levy JH. Coagulation abnormalities and thrombosis in patients with COVID-19. Lancet Haematol 2020;7:e438–40. 32407672 10.1016/S2352-3026(20)30145-9 PMC7213964

[4] Galván Casas C, Català A, Carretero Hernández G, Rodríguez-Jiménez P, Fernández-Nieto D, Rodríguez-Villa Lario A, et al. Classification of the cutaneous manifestations of COVID-19: a rapid prospective nationwide consensus study in Spain with 375 cases. Br J Dermatol 2020;183:71–7. 32348545 10.1111/bjd.19163 PMC7267236

[5] Perera TB, Murphy-Lavoie HM. Purpura fulminans. [Updated 21 July 2021]. In: StatPearls [Internet]. Treasure Island (FL): StatPearls Publishing; 2021. https://www.ncbi.nlm.nih.gov/books/NBK532865/. 30422460

[6] Parisi R, Costanzo S, Di Castelnuovo A, de Gaetano G, Donati MB, Iacoviello L. Different anticoagulant regimens, mortality, and bleeding in hospitalized patients with COVID-19: a systematic review and an updated meta-analysis. Semin Thromb Hemost 2021;47:372–91. 33851386 10.1055/s-0041-1726034

[7] Henry BM, Cheruiyot I, Benoit JL, Lippi G, Prohászka Z, Favaloro EJ, et al. Circulating levels of tissue plasminogen activator and plasminogen activator Inhibitor-1 are independent predictors of coronavirus disease 2019 severity: a prospective, observational study. Semin Thromb Hemost 2021;47:451–5. 33482678 10.1055/s-0040-1722308

[8] Favaloro EJ, Henry BM, Lippi G. Increased VWF and decreased ADAMTS-13 in COVID-19: creating a milieu for (micro)thrombosis. Semin Thromb Hemost 2021;47:400–18. 33893632 10.1055/s-0041-1727282

[9] Khan IA, Karmakar S, Chakraborty U, Sil A, Chandra A. Purpura fulminans as the presenting manifestation of COVID-19. Postgrad Med J 2021;97:473. 33563711 10.1136/postgradmedj-2020-139202 PMC7878050

